# Novel associations between *KCNQ1* rs231840 polymorphism and preeclampsia in Chinese gestational women: A case-control candidate genetic study

**DOI:** 10.1097/MD.0000000000039778

**Published:** 2024-10-11

**Authors:** Lingyu Ma, Rui Ma, Ran Ran, Jiaying Li, Xuefeng Pan, Zhiheng Guo, Xichen Lin, Dezhong Wen, Shuyao Wu, Ying Chen

**Affiliations:** a Department of Gynecology and Obstetrics Center, the First Hospital of Jilin University, Jilin, China; b Department of Obstetrics, Maternal and Child Health Hospital of Ningxia Hui Autonomous Region, Yinchuan, China; c Department of Research and Development, Yinfeng Biological Engineering Technology Company Limited, Jilin, China; d Affiliated Middle School to Jilin University, Jilin, China; e Department of Medical Genetics, College of Basic Medical Sciences, Jilin University, China.

**Keywords:** *KCNQ1*, polymorphism, preeclampsia

## Abstract

Preeclampsia is a complex disorder with genetic and environmental interactions. In this study, we analyzed the associations of *KCNQ1*gene polymorphisms with preeclampsia in Chinese pregnant women. The 3 candidate single-nucleotide polymorphisms rs231840, rs2237892, and rs2237895 were genotyped in this case-control study; clinical and biochemical data were included and SNPs were gathered from 248 individuals with preeclampsia and 237 controls. The TT genotype rs231840 increased the risk of preeclampsia (OR: 1.633; 95% CI: 1.027–2.597) and was associated with higher blood glucose levels. The haplotype TCA containing the allele of rs231840 (T), rs2237892 (C), and rs2237895 (A) was highly protective against preeclampsia and associated with the levels of blood glucose in preeclamptic patients. A novel function was found for the haplotype CCA in SNPs rs231840 (C), rs2237892 (C), and rs2237895 (A); it might be a protective combination against preeclampsia. The *KCNQ1* (TT) genotype seems to be associated with preeclampsia and might affect the regulation of blood glucose in Chinese pregnant women.

## 1. Introduction

Preeclampsia is traditionally detected by the presence of hypertension and proteinuria. Research on its pathogenesis emphasized the critical pathologic processes of endothelial cell activation, intravascular inflammation, and syncytiotrophoblast hypoxia/reoxygenation injury.^[1]^ The excessive decidual inflammation and reoxygenation injury in preeclampsia could be triggered by maternal obesity, diabetes mellitus, chronic hypertension, and autoimmune diseases.^[2]^ Our previous studies showed that dysregulated glycolipid metabolism contributed to the pathology of preeclampsia.^[3,4]^ We could perceive clues of glucose metabolism disorders in the development of those metabolic diseases, which seemed to share similar genetic backgrounds.

Genome-wide association studies (GWAS) data have also shown that Potassium Voltage-Gated Channel Subfamily Q Member 1 (*KCNQ1*) genes were candidate genes related to levels of fasting glucose and insulin secretion and sensitivity.^[5,6]^ The research on gene polymorphism can not only predict diseases, but also be applied to the field of targeted drug therapy.^[7]^ The *KCNQ1* gene, located in the region of chromosome 11p15.5, encodes a voltage-gated potassium channel and contributes to diverse physiological phenomena, including modulation of the membrane potential and neurotransmitter, hormone release, and electrolyte transport in epithelia.^[8]^ The rs2237892 and rs2237895 polymorphisms in the *KCNQ1* gene have been identified to be associated with type 2 diabetes (T2D) susceptibility in a meta-analysis that included over 70,000 T2D cases and 90,000 controls.^[9]^ The rs231357 polymorphism of the *KCNQ1* gene was also associated with DNA methylation and insulin sensitivity, and the latter condition might be involved in the pathology of preeclampsia.^[4,10,11]^ The methylation of the KCNQ1 gene has been linked to the level of insulin sensitivity and serum adiponectin levels, as revealed through a comparative population analysis driven by the SNP rs231840.^[12]^

The *KCNQ1* gene was an essential factor for proliferation inhibition in placentation and was involved in a response to an oxygenation stimulus.^[13,14]^ An increasing level of *KCNQ1* gene mRNA expression in placental tissue from preeclamptic women was also identified.^[15]^ It also played an essential role in regulating the vascular tone during the critical window of fetal-placental vascular development. Hence, it is the primary mechanism affecting perfusion in the placenta and vascular adaptation in patients with preeclampsia.^[16]^

Because it is still unknown whether those polymorphisms in the *KCNQ1* gene are associated with preeclampsia, we decided to investigate the relationship between the 3 SNPs rs231840, rs2237892, and rs2237895 and preeclampsia. A secondary study was performed to investigate the random blood glucose levels in Chinese pregnant women and the effects of genetic factors on these levels.

## 2. Methods and materials

### 2.1. Study design and population

This case-control study involved 485 pregnant women, 248 with preeclampsia and 237 controls, at the First Hospital of Jilin University from January 2019 to September 2021. All subjects were 16 to 45 years of age. All individuals from the Han population in Northeast China consented in writing to participate in the study. The research project was performed following the stipulations of the Declaration of Helsinki. Ethical approval was obtained from the local Ethics Committee of the First Hospital of Jilin University, Changchun, China (permission number: 2018-401). We had no conflicts with any medical advice or medications.

### 2.2. Inclusion and exclusion criterion

Preeclampsia was diagnosed based on the Gestational Hypertension and Preeclampsia: ACOG Practice Bulletin Summary, Number 222.^[17]^ Preeclampsia was defined as the new onset of hypertension and/or proteinuria (systolic blood pressure ≥ 140 mm Hg and/or diastolic blood pressure ≥ 90 mm Hg) after 20 weeks of gestation in a pregnant woman without previous hypertension. Severe hypertension is defined as systolic blood pressure of 160 mm Hg or higher and/or diastolic blood pressure of 110 mm Hg or higher, with or without significant end-organ dysfunction, persistent and/or severe headache, visual abnormalities, epigastric pain, or HELLP syndrome (hemolysis, elevated liver enzymes, low platelets).

The control group included normotensive pregnant females without a history of chronic hypertension, infection, hepatic or kidney diseases, gestational diabetes mellitus, heart failure, or systemic lupus erythematosus.

### 2.3. Data gathered on patients

General information, clinical parameters, and biochemical test results were obtained for women admitted to the hospital before delivery. Available characteristic information, including the patient’s age, weeks of pregnancy, body mass index before pregnancy (BMI = weight [kg]/height [m^2^]), twins, and use of assisted reproduction, a history of hypertension, was also noted. The blood pressure was measured by automated noninvasive devices twice at intervals of 30 minutes before hospitalization. Levels of the random blood glucose (RBG), thyroid-stimulating hormone (TSH), triiodothyronine (FT3), and thyroxine (FT4) were measured before delivery when the patient had had an empty stomach for more than 6 hours by an automatic biochemical analyzer (SEKISUI Medical Technology Ltd., Tokyo, Japan). A quantity of 3ml of venous blood was extracted and stored at –20°C for DNA extraction at the time of hospital admission.

### 2.4 DNA extraction and genotyping

DNA was extracted from leucocytes, and second-generation sequencing technology examined SNPs rs231840, rs2237892, and rs2237895. The detection method was the same as used in previous research studies.^[3,4]^

### 2.5. Statistical analysis

Based on Wayne W’s advice in medical statistics,^[18]^ patients were divided into 2 groups based on the diagnostic criteria. Among the parameters, twins, assisted reproduction, and a history of hypertension, as well as genotype and allele information, were statistically described as categorical data.^[19]^ For continuous variables such as patient’s age, weeks of pregnancy, BM, RBG, TSH, FT3, and FT4, statistical descriptions were performed separately after the normality test.^[19]^

To control for the confounding effects of multiple factors on genetic susceptibility to disease, regression analysis was utilized. Through univariate analysis, significant independent variables were identified and selected for inclusion in the regression model. Within this regression analysis, further investigation was undertaken to comprehend how these independent variables collectively and individually influence the dependent variable, with their respective impacts being quantitatively assessed.

Statistical analysis was performed using SPSS Version 18.0 (SPSS Inc., Chicago). All the continuous variables with non-normal distribution were represented as the median (inter quartile range), and the *U* test was used to assess the differences between the control and preeclampsia groups. The Hardy–Weinberg equilibrium was calculated, and the differences in the distribution of the alleles and genotypes were determined using information found at https://www.snpstats.net/start.htm?. Odds ratios (ORs) with 95% confidence intervals (CIs) were calculated using a logistic regression model to determine the odds of developing preeclampsia. A *P* value of <.05 was considered a significant difference.

## 3. Results

The epidemiological data are shown in Table 1. All the continuous variables were examined and identified as non-normal distributions. Compared with the controls, the patients with preeclampsia were found to have significantly higher systolic blood pressure measurements, BMIs before pregnancy, and levels of RBG, TSH, and FT4, and the gestational weeks, diastolic blood pressure, and FT3 were much lower in the patients with preeclampsia. Moreover, the use of assisted reproduction, twins, and a history of hypertension were noticeably more common in the patients with preeclampsia than in the controls. The characteristics of lipid metabolism, thyroid function, and BMI had been noted in previous reports, and the results were similar.^[3,4,20]^ The RBG level was obtained in patients who had had an empty stomach for over 6 hours.

**Table 1:** The clinical and demographic data of pregnant women with PE.

Variables	Controls (N = 237)	PE (N = 248)	*P* value
Maternal age (yr)	30.00 (28.00–33.00)	31.00 (28.00–35.00)	.083
AG (wk)	39.00 (38.43–39.71)	35.00 (31.93–37.29)	<.001*
Assisted reproduction	11 (4.6%)	27 (10.9%)	.011*
Twins	6 (2.5%)	15 (6.0%)	.057
History of hypertension	0 (0%)	11 (4.4%)	.001*
Systolic blood pressure (mm Hg)	117.00 (110.00–123.00)	170.00 (160.00–180.00)	<.001*
Diastolic blood pressure (mm Hg)	78.00 (72.00–82.00)	110.00 (105.00–120.00)	<.001*
BMI before pregnancy	21.09 (19.28–23.14)	23.44 (21.27–27.04)	<.001*
RBG (mmol/L)	4.35 (3.90–4.95)	4.64 (4.13–5.50)	<.001*
TSH (uIU/mL)	1.97 (1.36–2.68)	3.24 (2.18–4.91)	<.001*
FT3 (pmol/L)	4.59 (3.95–5.14)	3.96 (3.47–4.53)	<.001*
FT4 (pmol/L)	10.40 (9.64–11.67)	11.15 (9.82–13.01)	.008*

AG = age of gestation, BMI = body mass index, FT3 = free triiodothyronine, FT4 = free thyroxine, PE = preeclampsia, RBG = random blood glucose, TSH = thyroid-stimulating hormone.

* *P* < .05.

All the genotype frequencies are shown in Table 2. The distribution of the genotypes followed the Hardy–Weinberg equilibrium (*P* > .05), and the results are shown in the supplementary information in Table S1, Supplemental Digital Content. http://links.lww.com/MD/N628

**Table 2:** Analysis of association between genotype and preeclampsia.

Genotypes	Controls (N = 237)	PE (N = 248)	OR (95% CI)	*P* value
rs231840				
Codominant T/T	138 (58.2%)	162 (65.3%)	1.00	
C/T	87 (36.7%)	79 (32.0%)	0.77 (0.53–1.13)	
C/C	12 (5.1%)	7 (2.8%)	0.50 (0.19–1.30)	.18
Dominant T/T	138 (58.2%)	162 (65.3%)	1.00	
C/T–C/C	99 (41.8%)	86 (34.7%)	0.74 (0.51–1.07)	.11
Recessive T/T–C/T	225 (94.9%)	241 (97.2%)	1.00	
C/C	12 (5.1%)	7 (2.8%)	0.54 (0.21–1.41)	.20
Overdominant T/T–C/C	150 (63.3%)	169 (68.2%)	1.00	
C/T	87 (36.7%)	79 (31.9%)	0.81 (0.55–1.17)	.26
rs2237892				
Codominant C/C	108 (45.6%)	116 (47.0%)	1.00	
C/T	97 (40.9%)	94 (38.0%)	0.90 (0.61–1.33)	
T/T	32 (13.5%)	38 (15.0%)	1.11 (0.65–1.89)	.74
Dominant C/C	108 (45.6%)	116 (46.8%)	1.00	
C/T–T/T	129 (54.4%)	132 (53.2%)	0.95 (0.67–1.36)	.79
Recessive C/C–C/T	205 (86.5%)	210 (84.7%)	1.00	
T/T	32 (13.5)	38 (15.3%)	1.16 (0.70–1.93)	.57
Overdominant C/C–T/T	140 (59.1%)	154 (62.1%)	1.00	
C/T	97 (40.9%)	94 (37.9%)	0.88 (0.61–1.27)	.50
rs2237895				
Codominant A/A	120 (50.6)	106 (43.0%)	1.00	
A/C	91 (38.4%)	110 (44.0%)	1.37 (0.93–2.00)	
C/C	26 (11.0%)	32 (13.0%)	1.39 (0.78–2.49)	.22
Dominant A/A	120 (50.6%)	106 (42.7%)	1.00	
A/C–C/C	117 (49.4%)	142 (57.3%)	1.37 (0.96–1.97)	.082
Recessive A/A–A/C	211 (89%)	216 (87.1%)	1.00	
C/C	26 (11%)	32 (12.9%)	1.20 (0.69–2.09)	.51
Overdominant A/A–C/C	146 (61.6%)	138 (55.6%)	1.00	
A/C	91 (38.4%)	110 (44.4%)	1.28 (0.89–1.84)	.18

CI = confidence interval, OR = odds ratio.

*P < .05 defined as statistically significant.

The distribution of the genotypes of SNPs of the *KCNQ1* gene (rs231840, rs2237892, and rs2237895) among the patients is shown in Table 2. There was no association between the genotype and preeclampsia on single-factor analysis when a *P* value of <.05 was defined as statistically significant.

*KCNQ1* gene (rs231840, rs2237892, and rs2237895) haplotype analysis. To investigate the association between the *KCNQ1* gene SNPs and preeclampsia, we analyzed the haplotypes of the *KCNQ1* gene and found a linkage disequilibrium (*r*^2^ = 0.196; *D*′ = 0.8833). Then, we further determined which allele combination from the 3 SNPs was associated with preeclampsia. Table 3 shows the association between haplotype frequency and preeclampsia. The C(rs231840)–C(rs2237892)–A(rs2237895) haplotype combination had frequencies ranging from 12.19% down to 6.47% in the control group compared with the preeclampsia group. The haplotype combination C–C–A bestowed an effect against the development of preeclampsia (*P* = .026, 0.51 [0.28–0.92]).

**Table 3:** Haplotype frequency and association with preeclampsia for *KCNQ1*.

rs231840	rs2237892	rs2237895	Frequency	OR (95% CI)	*P* value
T	T	A	0.2666	1.00	–
T	C	C	0.2648	1.15 (0.79–1.66)	.47
T	C	A	0.2512	1.04 (0.71–1.52)	.86
C	C	A	0.0934	0.51 (0.28–0.92)	.026*
C	T	A	0.062	1.07 (0.51–2.23)	.86
C	C	C	0.0494	1.41 (0.63–3.14)	.4
T	T	C	0.007	1.07 (0.27–4.16)	.93†

CI = confidence interval, OR = odds ratio.

* *P* < .05 defined as statistically significant.

† OR was co-calculated for rare haplotype combination.

Binary logistic regression analysis. We used binary logistic regression analysis for the risk factors involved, and the involved criteria were designated as having a *P* value of <.20 as calculated by single-factor analysis. All the risk parameters, including the SNPs, age of the patient, BMI before pregnancy, history of the patient, and TSH levels, were involved in the regression model. The results showed that rs231840 (TT), the BMI, the TSH levels, a history of chronic hypertension, and the use of assisted reproduction were associated with preeclampsia.

Table 4 shows the relationships between the risk parameters and preeclampsia. The area under curve (AUC) of the receiver operating characteristic (ROC) curve was 0.842 (*P* < .001, 95% CI: 0.808–0.876) based on the above parameters, and it might be used to predict the risk of preeclampsia.

**Table 4:** Odds ratios (95% confidence intervals) for the association between PE and different parameters.

Parameters	*B*	*P* value	OR	95% CI for EXP (B)	
				Lower	Upper
rs231840 (TT)	0.519	.028*	1.680	1.057	2.671
Assisted reproduction	0.998	.017	2.714	1.194	6.170
History of chronic hypertension	2.230	.040	9.297	1.109	77.953
BMI before pregnancy	0.221	.000*	1.247	1.169	1.331
TSH (uIU/mL)	0.679	.000*	1.972	1.662	2.339
RBG (mmol/L)	0.342	.001*	1.408	1.152	1.720

BMI = body mass index, CI = confidence interval, OR = odds ratio, PE = preeclampsia, RBG = random blood glucose, TSH = thyroid-stimulating hormone.

* *P* < .05 defined as statistically significant.

We further investigated the relationships between the RBG and the genotypes in the preeclampsia group and found that the pregnant women with the T/T (rs231840) or A/C–A/A (rs2237895) genotypes had higher levels of RBG (Table 5).

**Table 5:** The relationships between RBG and genotypes.

Parameters	Genotypes	RBG (mmol/L)	*P* value
rs231840 Codominant (N = 248)	T/T (N = 162)	4.72 (4.18–5.55)	.015*
	C/T (N = 79)	4.64 (4.11–5.15)	
	C/C (N = 7)	3.89 (3.54–4.33)	
Dominant	T/T (N = 162)	4.72 (4.18–5.55)	.045*
	C/T + C/C (N = 86)	4.55 (3.99–5.11)	
rs2237892 Codominant (N = 248)	C/C (N = 116)	4.72 (4.17–5.54)	<.001*
	C/T (N = 94)	4.86 (4.18–5.58)	
	T/T (N = 38)	4.25 (3.84–4.70)	
Dominant	C/C (N = 116)	4.72 (4.17–5.54)	.118
	C/T + T/T (N = 132)	4.55 (4.06–5.23)	
rs2237895 Codominant (N = 248)	A/A (N = 106)	4.42 (3.93–5.12)	.009*
	A/C (N = 110)	4.77 (4.20–5.49)	
	C/C (N = 32)	5.14 (4.50–5.87)	
Dominant (N = 248)	A/A (N = 106)	4.42 (3.93–5.12)	.006*
	A/C–C/C (N = 142)	4.82 (4.21–5.58)	

RBG = random blood glucose.

* *P* < .05 defined as statistically significant.

All the preeclamptic pregnant women were divided into 2 subgroups: those with RBG levels of 5.1 mmol/L or higher (n = 88 women) and those with RBG levels of <5.1 mmol/L (n = 160 women). A haplotype analysis of the RBG subgroup showed that combinations of the haplotypes TTA, CCA, and CCC were present more frequently in preeclamptic women with lower RBG levels (Table 6).

**Table 6:** Haplotype frequency and association with RBG in preeclampsia for *KCNQ1* (n = 248).

rs231840	rs2237892	rs2237895	Frequency	OR (95% CI)	*P* value
T	C	C	0.2821	1.00	–
T	C	A	0.26	0.69 (0.39–1.22)	.2
T	T	A	0.2567	0.29 (0.16–0.53)	1e-04*
C	T	A	0.0723	0.55 (0.23–1.31)	.18
C	C	A	0.0603	0.19 (0.04–0.83)	.028*
C	C	C	0.0549	0.18 (0.03–0.92)	.041*
T	T	C	0.0138	0.69 (0.10–4.94)	.71

CI = confidence interval, OR = odds ratio, RBG = random blood glucose.

* *P* < .05 defined as statistically significant.

## 4. Discussion

In this study, we first reported that the *KCNQ1* gene SNP rs231840 became associated with preeclampsia by influencing the blood glucose levels. The haplotype combination CCA was a protective factor for preeclampsia and one of 3 advantageous haplotype combinations (TTA, CCC, and CCA) associated with lower RBG levels. We speculated that the polymorphism of the *KCNQ1* gene was an essential hereditary parameter influencing the pathology of preeclampsia by regulating the level of the blood glucose.

*KCNQ1* is also known to be a voltage-dependent K+ channel that regulates glucose homeostasis.^[21]^ SNP rs231840 could drive methylation at the *KCNQ1* gene locus by the polymorphically substitution of a cytosine residue within a CpG site.^[22]^ A study cohort focused on the “Relationship between Insulin Sensitivity and Cardiovascular Disease” (RISC) revealed that the single nucleotide polymorphism (SNP) rs231840 serves as a robust predictor for the methylation status of rs231840, whereas this methylation pattern was not found to correlate with any other metabolic indicators in a European population.^[12]^

The KCNQ1 gene SNP rs231840 might be associated with preeclampsia potentially, and this hypothesis could be tested through binary logistic regression analysis. However, it is important to note that in the current study, the *P* value obtained from single-factor analysis was <.05, indicating statistical significance but requiring further validation through multivariate analysis or replication in larger studies. In diabetes patients, the levels of DNA methylation with the rs231840 TT genotype were significantly lower than with the other variant alleles.^[12]^ The level of methylation of the KCNQ1 gene imprinted gametic differentially methylated domains, as a key regulator of placental function, was associated with trophoblastic giant cell expansion.^[23]^ We have not learned how the *KCNQ1* gene influences the onset of preeclampsia, but we did find that it played an essential role in controlling the vascular tone and responses to altered oxygenation.^[15,22]^

Besides influencing DNA methylation, the SNP rs231840 also affects insulin sensitivity.^[12]^ Animal studies in gene-targeted mice revealed that the *KCNQ1* gene knockout resulted in enhanced insulin sensitivity and that increased *KCNQ1* protein expression could limit insulin secretion in pancreatic beta cells. This is, in turn, critically important for the regulation of positive feedback in glucose metabolism by influencing K+-dependent insulin signaling. The expression of both placental growth factor and vascular endothelial growth factor was significantly higher in choriocarcinoma cells in vivo conditions with high blood glucose levels.^[24]^ Abnormal glucose metabolism is a significant risk factor for preeclampsia because it disrupts the balance of placental angiogenic factors.^[25]^ In our study, the pregnant women with the rs231840 TT genotype had higher blood glucose levels and also a greater risk for preeclampsia.

It was observed that the haplotype CCA containing the allele of rs231840 (C), rs2237892 (C), and rs2237895 (A) was highly protective against preeclampsia and decreased RBG levels in those women with preeclampsia. There was a linkage disequilibrium in the 3 SNPs rs231840, rs2237892, and rs2237895 in the *KCNQ1*gene. The haplotypes rs2237892 (T) and rs2237895 (A) were highly protective against T2D in a previous report,^[26]^ which also showed the protective associations of RBG with preeclampsia in our study. Among those haplotypes, CCA also showed a protective effect against preeclampsia in Chinese pregnant women, although we did not find the mechanism of the pathophysiology. Further studies could build on our findings and investigate the polymorphism of the *KCNQ1* gene and gene expression levels.

## 5. Conclusions

A novel function was found and indicated that the haplotypes CCA in SNPs rs231840 (C), rs2237892 (C), and rs2237895 (A) might form a protective combination against preeclampsia. *KCNQ*1 gene polymorphisms are associated with preeclampsia and might affect the regulation of blood glucose levels in Chinese pregnant women.

### 5.1. Limitations

The number of people included in this study is limited, and more stable conclusions will be obtained after expanding the number of study participants. Another limitation of this article is that it ignores the impact of environmental factors on disease and fails to incorporate patients’ lifestyle factors in experimental design, resulting in a lack of research on the interaction between genes and human behavior habits. Despite these limitations, the study demonstrates that the haplotypes CCA in SNPs rs231840 (C), rs2237892 (C), and rs2237895 (A) might form a protective combination against preeclampsia.

## Acknowledgments

We confirm that the manuscript has been read and approved by all named authors and that there are no other persons who satisfied the criteria for authorship but are not listed. We further confirm that the order of authors listed in the manuscript has been approved by all of us.

## Author contributions

**Conceptualization:** Xuefeng Pan, Dezhong Wen, Ying Chen.

**Data curation:** Ran Ran, Xichen Lin.

**Formal analysis:** Rui Ma, Ran Ran, Zhiheng Guo.

**Funding acquisition:** Ying Chen.

**Software:** Jiaying Li.

**Writing – original draft:** Lingyu Ma, Shuyao Wu.

**Writing – review & editing:** Xuefeng Pan, Zhiheng Guo, Ying Chen.

## Supplementary Material



## References

[1] Jung E Romero R Yeo L. The etiology of preeclampsia. Am J Obstet Gynecol. 2022;226(2S):S844–66. 35177222 10.1016/j.ajog.2021.11.1356 PMC8988238

[2] Staff AC Dechend R Redman CW. Review: preeclampsia, acute atherosis of the spiral arteries and future cardiovascular disease: two new hypotheses. Placenta. 2013;34(Suppl):S73–8. 23246096 10.1016/j.placenta.2012.11.022

[3] Pan X Wei B Wang H Ma L Du Z Chen Y. Novel association between FOXO3 rs2232365 polymorphism and late-onset preeclampsia: a case-control candidate genetic study. BMC Pregnancy Childbirth. 2020;20:779. 33317466 10.1186/s12884-020-03479-6 PMC7737381

[4] Wang H Ma L Pan X Du Z Chen Y. Novel associations of SNPs MYLIP rs3757354 and ABCA1 2230806 gene with early-onset-preeclampsia: a case-control candidate genetic study. Pregnancy Hypertension. 2020;23:185–90. 33450693 10.1016/j.preghy.2020.12.005

[5] Lahrouchi N Tadros R Crotti L. Transethnic genome-wide association study provides insights in the genetic architecture and heritability of long QT syndrome. Circulation. 2020;142:324–38. 32429735 10.1161/CIRCULATIONAHA.120.045956 PMC7382531

[6] Voight BF Scott LJ Steinthorsdottir V.; MAGIC investigators. Twelve type 2 diabetes susceptibility loci identified through large-scale association analysis. Nat Genet. 2010;42:579–89. 20581827 10.1038/ng.609 PMC3080658

[7] Pearce B Abrahams-October Z Xhakaza L Jacobs C Benjeddou M. Effect of the African-specific promoter polymorphisms on the SLC22A2 gene expression levels. Drug Metab Pers Ther. 2018;33:85–9. 29624501 10.1515/dmpt-2017-0039

[8] Kuenze G Duran AM Woods H. Upgraded molecular models of the human KCNQ1 potassium channel. PLoS One. 2019;14:e0220415. 31518351 10.1371/journal.pone.0220415 PMC6743773

[9] Sun Q Song K Shen X Cai Y. The association between KCNQ1 gene polymorphism and type 2 diabetes risk: a meta-analysis. PLoS One. 2012;7:e48578. 23133642 10.1371/journal.pone.0048578 PMC3487731

[10] Chen Z Yin Q Ma G Qian Q. KCNQ1 gene polymorphisms are associated with lipid parameters in a Chinese Han population. Cardiovasc Diabetol. 2010;9:35. 20701788 10.1186/1475-2840-9-35 PMC2925337

[11] Zaydman MA Cui J. PIP2 regulation of KCNQ channels: biophysical and molecular mechanisms for lipid modulation of voltage-dependent gating. Front Physiol. 2014;5:195. 24904429 10.3389/fphys.2014.00195 PMC4034418

[12] Shah UJ Xie W Flyvbjerg A.; RISC consortium. Differential methylation of the type 2 diabetes susceptibility locus KCNQ1 is associated with insulin sensitivity and is predicted by CpG site specific genetic variation. Diabetes Res Clin Pract. 2019;148:189–99. 30641161 10.1016/j.diabres.2019.01.008 PMC6395844

[13] Chan PJ Osteen JD Xiong D. Characterization of KCNQ1 atrial fibrillation mutations reveals distinct dependence on KCNE1. J Gen Physiol. 2012;139:135–44. 22250012 10.1085/jgp.201110672 PMC3269792

[14] Cosme D Estevinho MM Rieder F Magro F. Potassium channels in intestinal epithelial cells and their pharmacological modulation: a systematic review. Am J Physiol Cell Physiol. 2021;320:C520–46. 33326312 10.1152/ajpcell.00393.2020 PMC8424539

[15] Mistry HD Kurlak LO Whitley GS Cartwright JE Broughton Pipkin F Tribe RM. Expression of voltage-dependent potassium channels in first trimester human placentae. Placenta. 2014;35:337–40. 24646441 10.1016/j.placenta.2014.02.008

[16] Morley LC Debant M Walker JJ Beech DJ Simpson NAB. Placental blood flow sensing and regulation in fetal growth restriction. Placenta. 2021;113:23–8. 33509641 10.1016/j.placenta.2021.01.007 PMC8448138

[17] Gestational hypertension and preeclampsia: ACOG practice bulletin summary, number 222. Obstet Gynecol. 2020;135:1492–5. 32443077 10.1097/AOG.0000000000003892

[18] Daniel WW. Biostatistics: A Foundation for Analysis in the Health Sciences. 7th ed. New York: Wiley; 1999. xiv+755+appendices.

[19] Elston RC Johnson WD Hokanson JE. Basic biostatistics for geneticists and epidemiologists: a practical approach. Am J Epidemiol. 2009;170:130.

[20] Song W Wang H Ma L Chen Y. Associations between the TNMD rs4828038 and ACE2 rs879922 polymorphisms and preeclampsia susceptibility: a case-control study. J Obstet Gynaecol. 2022;42:1132–6. 34996340 10.1080/01443615.2021.2012438

[21] Sun J MacKinnon R. Structural basis of human KCNQ1 modulation and gating. Cell. 2020;180:340–7.e9. 31883792 10.1016/j.cell.2019.12.003 PMC7083075

[22] Jung SY Bhatti P Pellegrini M. DNA methylation in peripheral blood leukocytes for the association with glucose metabolism and invasive breast cancer. Clin Epigenetics. 2023;15:23. 36782224 10.1186/s13148-023-01435-7 PMC9926571

[23] Koppes E Himes KP Chaillet JR. Partial loss of genomic imprinting reveals important roles for Kcnq1 and Peg10 imprinted domains in placental development. PLoS One. 2015;10:e0135202. 26241757 10.1371/journal.pone.0135202 PMC4524636

[24] Mitsui T Tani K Maki J. Upregulation of angiogenic factors via protein kinase C and hypoxia-induced factor-1α pathways under high-glucose conditions in the placenta. Acta Med Okayama. 2018;72:359–67. 30140083 10.18926/AMO/56171

[25] Burton GJ Cindrova-Davies T Yung HW Jauniaux E. Hypoxia and reproductive health: oxygen and development of the human placenta. Reproduction. 2021;161:F53–65. 32438347 10.1530/REP-20-0153

[26] Turki A Mtiraoui N Al-Busaidi AS Khirallah M Mahjoub T Almawi WY. Lack of association between genetic polymorphisms within KCNQ1 locus and type 2 diabetes in Tunisian Arabs. Diabetes Res Clin Pract. 2012;98:452–8. 23107108 10.1016/j.diabres.2012.10.006

