# Supplementary material for: Novel associations between KCNQ1 rs231840 polymorphism and preeclampsia in Chinese gestational women: A case-control candidate genetic study
Source: Medicine (Baltimore). 2024 Oct 11;103(41):e39778. doi: 10.1097/MD.0000000000039778 (PMC11479463; doi:10.1097/MD.0000000000039778)
Supplement: Supplementary file 1 [file medi-103-e39778-s001.docx]

Table S1. Hardy-weinberg equilibrium testing.

|  | Genotype | | | Allele | | *P* Value |
| --- | --- | --- | --- | --- | --- | --- |
| rs231840 | T/T | C/T | C/C | T (%) | C (%) |  |
| Controls (N = 237) | 138 | 87 | 12 | 363 | 111 | 0.86 |
| PE (N = 248) | 162 | 79 | 7 | 403 | 93 | 0.68 |
| rs2237892 | C/C | C/T | T/T | C | T |  |
| Controls (N = 237) | 108 | 97 | 32 | 313 | 161 | 0.19 |
| PE (N = 248) | 116 | 94 | 38 | 326 | 170 | 0.016 |
| rs2237895 | A/A | A/C | C/C | A | C |  |
| Controls (N = 237) | 120 | 91 | 26 | 331 | 143 | 0.17 |
| PE (N = 248) | 106 | 110 | 32 | 322 | 174 | 0.68 |

*P*＞0.05 indicates that it conforms to Hardy-weinberg equilibrium.
